# Mechanistic Insights and Emerging Therapeutic Targets of Alzheimer's Disease: From the Perspective of Inter-Organ Crosstalk

**DOI:** 10.14336/AD.2024.1499

**Published:** 2025-12-28

**Authors:** Jianqiong Yin, Wei Peng, Lu Lu, Zhen Hong, Dong Zhou, Jinmei Li

**Affiliations:** Department of Neurology, West China Hospital of Sichuan University, Chengdu, Sichuan 610061, China

**Keywords:** Alzheimer’s disease;, brain, inter-organ crosstalk, mechanism

## Abstract

With complex pathogenesis, Alzheimer's disease (AD) is a neurological illness that has worsened over time. Inter-organ crosstalk, which is essential for coordinating organ function and maintaining homeostasis, is involved in multiple physiological and pathological events. Increasing evidence suggests that AD is closely associated with multiple diseases of peripheral organs, including the gut, adipose tissue, liver, and bone. Despite numerous studies on AD, the ambiguous role of pathological peripheral organ-brain crosstalk in the development of AD remains incompletely understood, and the potential mechanisms remain obscure. This review summarizes the current knowledge of the relationship between AD and disorders of various organs from clinical and preclinical evidence. Additionally, we elucidate the mechanisms underlying AD development from the perspective of “organ-organ crosstalk”, including the gut-brain, adipose tissue-brain, liver-brain and bone-brain axes. On the basis of the peripheral organ-brain crosstalk, we emphasize promising therapeutic targets with the hope of providing novel perspectives for AD management.

## 1. Introduction

Alzheimer’s disease (AD) is a neurodegenerative disorder and the main cause of dementia in elderly individuals. Currently, 50 million individuals are vulnerable to AD globally according to the estimation of Alzheimer's Disease International, and it is estimated that the incidence of AD will increase by 2 times by 2050 [1]. The primary clinical features of AD include cognitive deterioration, difficulties in daily activities and neuropsychiatric symptoms [2]. The pathological hallmarks of AD include the formation of amyloid plaques and neurofibrillary tangles, resulting from the accumulation of amyloid beta (Aβ) and tau proteins [3]. Current therapeutic strategies, including acetylcholinesterase inhibitors and N-methyl-D-aspartate receptor antagonists, are largely ineffective and cannot prevent or cure the disease [4]. The development of effective therapies demands a deep comprehension of the key factors driving the progression of AD.

Currently, inter-organ crosstalk is pivotal for maintaining human homeostasis and disease pathogenesis [5]. Most organs can communicate with local and distant organs through the secretion of lipids, proteins, metabolites and extracellular vesicles in an endocrine/paracrine/autocrine manner [6]. Emerging evidence suggests that peripheral organ-brain crosstalk is central for brain health and neurological diseases, such as AD [7, 8]. Studies highlight that AD is strongly linked to other organ disorders, including gut dysbiosis, obesity, hepatic dysfunction, and orthopedic disease, as shown in Table 1 [9-12]. The brain and peripheral organs, like the gut, adipose tissue, liver, and bone, can communicate in various ways through the gut-brain axis, adipose tissue-brain axis, liver-brain axis, and bone-brain axis, consequently influencing the onset and progression of AD [13-15]. Thus, a deep understanding of the peripheral organ-brain crosstalk is critical for the early diagnosis and treatment of AD and the exploration of potential novel therapeutic targets.

In this review, we summarize the peripheral organ disorders associated with AD and relevant clinical and preclinical evidence. We also highlight the contribution of peripheral organ-brain crosstalk to the pathological processes of AD and elucidate promising therapeutic targets.

**Table 1:** The organ disorders associated with AD.

Organ	Disorder
**Gut**	Gut dysbiosis Inflammatory bowel disease Irritable bowel syndrome Celiac disease
**Adipose tissue**	Obesity
**Liver**	Cirrhosis Hepatitis virus infection Increased AST/ALT ratio Nonalcoholic fatty liver disease
**Bone**	Osteoporosis Osteoarthritis Rheumatoid arthritis

Abbreviations: AD: Alzheimer's disease; AST: aspartate aminotransferase; ALT: alanine aminotransferase.

## 2. Crosstalk between the brain and other organs in the development of AD

The underlying mechanisms of AD progression are complex and multifactorial. It is becoming increasingly clear that AD is not unique to the brain and involves multiple extracerebral organs [16]. In recent years, multiple animal and clinical studies have revealed that the pathogenesis of AD is closely associated with multiple organ disorders, including gut dysbiosis, metabolic disorders, hepatic diseases, and orthopedic diseases [17-19]. Emerging evidence suggests that brain-centered inter-organ crosstalk is pivotal in AD, but the underlying mechanisms of inter-organ crosstalk involved in AD development have yet to be fully elucidated.

## 2.1 Gut-brain crosstalk

### 2.1.1 Evidence for the involvement of intestinal disorders in AD development

Crosstalk between the brain and gut occurs via the so-called gut-brain axis, which is a complex crosstalk network involving several signaling pathways, including the vagus nerve, immune and neuroendocrine pathways [20]. AD is influenced mainly by genetic, lifestyle and environmental factors [21, 22]. And AD is frequently accompanied by intestinal disorders, indicating the important role of intestinal disorders in AD pathology [23]. Emerging clinical and preclinical data confirm the involvement of intestinal disorders in the pathogenesis of AD [24]. In a previous study, Cattaneo et al. revealed that patients with cognitive dysfunction had a greater abundance of the proinflammatory gut microbiome and a reduced presence of the anti-inflammatory gut microbiome, which was notably associated with the production of a peripheral inflammatory state [25]. Recent research has suggested that the fecal microbial diversity of AD patients is decreased, along with reduced *phylum Firmicutes* and highly enriched *Proteobacteria*. The proportion of changed microbiomes and the clinical severity of AD are strongly correlated [20]. AD patients with gut dysbiosis had increased *Actinobacteria* and decreased *Bacteroidetes* [26]. Numerous clinical studies have demonstrated a link between AD and multiple intestinal disorders, including inflammatory bowel disease [27, 28], irritable bowel syndrome [29] and celiac disease [30]. Disorders of the gut are also found in various AD mouse models [31]. Chen et al. suggested that dysbiosis of the gut is involved in amyloid pathology in AD mice [32]. Notably, Aβ precursor protein (APP)-transgenic mice lacking the gut microbiota exhibited diminished Aβ deposition in comparison to their counterparts with intestinal microbiota [33].

### 2.1.2 The potential mechanisms of gut-brain crosstalk in the development of AD

The underlying mechanisms of intestinal disorders in AD pathogenesis involve disruption of the intestinal barrier and blood-brain barrier (BBB), immune dysregulation, neuroinflammation, amyloid and tau aggregation, impaired neuronal excitability, altered neurogenesis and synaptic plasticity.

Intestinal disorders related to AD pathogenesis include reduced variation in the microbial community and compositional shifts, such as increases in *Escherichia, Bacteroidetes* and *Shigella spp*. and decreases in *Firmicutes* and *Bifidobacterium spp*. [25, 34]. Dysbiotic gut microbiota subsequently produce and secrete metabolites, functional byproducts and toxins such as short-chain fatty acids (SCFAs), amino acids, bile acids, neurotransmitters, lipopolysaccharides (LPS), polysaccharide A (PSA) and amyloids, which enter the circulatory system through a disrupted intestinal barrier and a leaky gut [35-37]. LPS activates the downstream nuclear factor kappa beta (NF-κB) pathway by binding to Toll-like receptor 4 (TLR4) receptors of various immune cells, including peripheral macrophages, neutrophils, dendritic cells, monocytes and brain microglia, to induce the secretion of proinflammatory cytokines, such as interleukin-1 (IL-1), IL-6, IL-1β and tumor necrosis factor α (TNF-α) [38, 39]. These cytokines can induce Aβ production by boosting β-secretase 1 (BACE1) enzyme activity and increasing the mRNA levels of APP [40]. The BACE1 enzyme is vital for Aβ production and cleaves the β site of APP to generate the Aβ protein [41]. LPS can also interfere with microglial functions via activating NF-κB signaling to induce the transcription of proinflammatory miRNAs [42]. For instance, miRNA-34a inhibits the phagocytosis of Aβ by microglia, thereby promoting AD progression [43]. In addition, LPS contributes to increasing BBB permeability, which promotes easier entry of proinflammatory substances into the central nervous system (CNS) while reducing the excretion of Aβ from the brain [44, 45]. Glycogen synthase kinase 3β (GSK-3β) is a major kinase related to tau hyperphosphorylation [46]. Research has shown that LPS can also induce GSK-3β phosphorylation via inflammatory cytokines, subsequently mediating tau hyperphosphorylation and aggregation [47].

**Figure 1.**
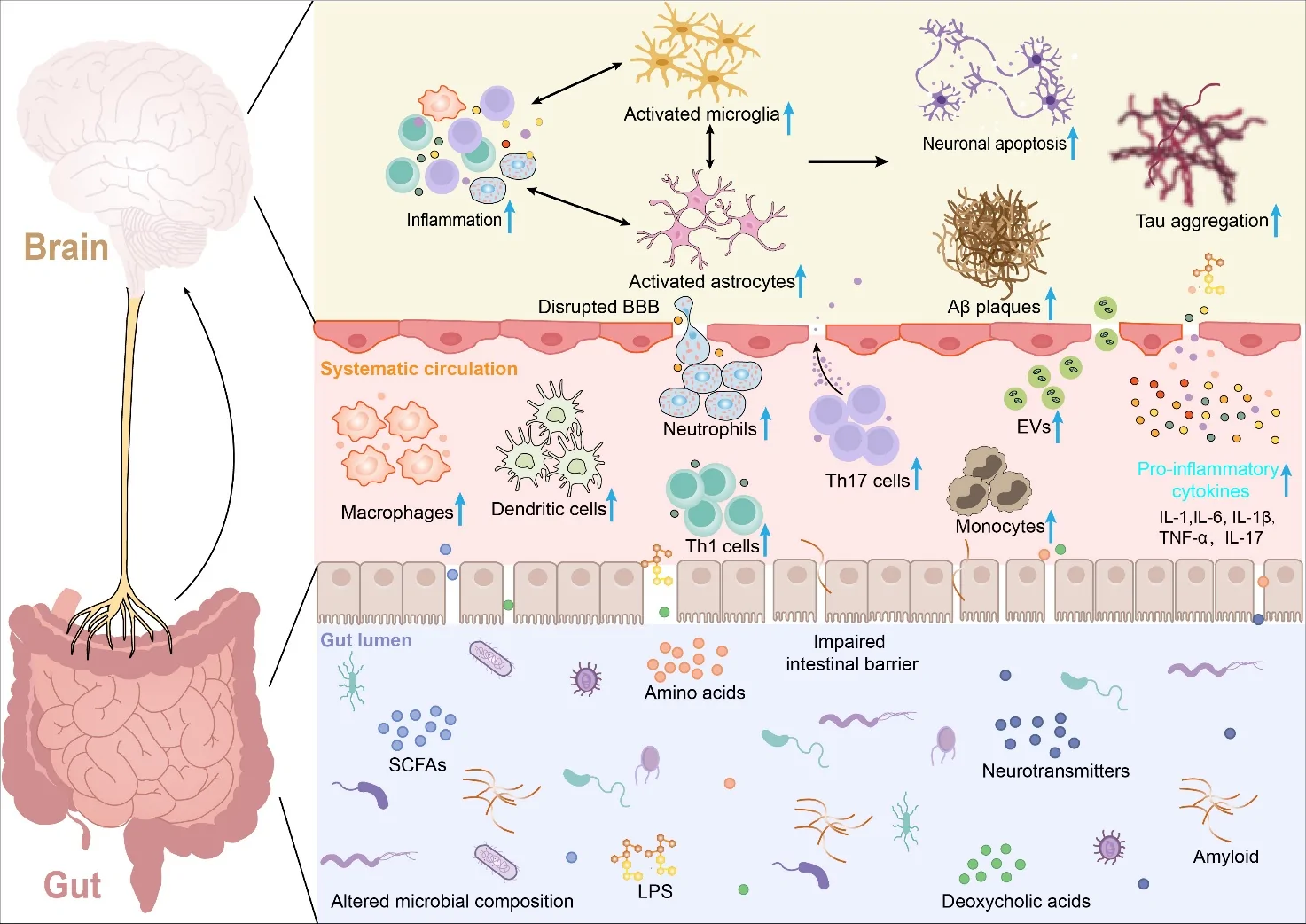
**Mechanisms of gut-brain crosstalk in the development of AD**. Crosstalk between the brain and gut occurs through the gut-brain axis, which is a complex crosstalk network that participates in the pathogenesis and progression of AD through a variety of pathways. Gut dysbiosis leads to an increase in a remarkably complex array of metabolites, functional byproducts and toxins such as SCFAs, amino acids, bile acids, neurotransmitters, LPS and amyloids. These changes induce systemic inflammatory and neuroinflammatory responses, including the activation of various immune cells, microglia and astrocytes and the secretion of inflammatory cytokines, which can then modulate tauopathy, amyloidosis and neurodegeneration, thus contributing to the development of AD.

Colombo et al. reported that gut microbiota-derived SCFAs promoted Aβ deposition via modulating the microglial phenotype [48]. The gut microbiome induces more conversion of bile acids into deoxycholic acid, and deoxycholic acid disrupts the BBB and enters the brain to induce AD [49]. Amino acids derived from the gut microbiome, such as isoleucine and phenylalanine, stimulate proinflammatory T helper 1 (Th1) cell proliferation, contributing to the AD-related neuroinflammatory response through the activation of M1 microglia [50]. Notably, gut dysbiosis can promote intestinal Th17 cell polarization followed by increased secretion of IL-17, inducing oligodendrocyte and neuronal apoptosis, neuroinflammation via microglial/astrocytic activation and neurovascular uncoupling, eventually leading to AD progression [51, 52]. Amyloids, bacterial byproducts produced by a variety of gram-positive and gram-negative bacteria, like *Proteobacteria*, *Actinobacteria*, *Bacteroidetes*, and *Firmicutes*, also contribute to AD pathogenesis [53, 54]. Studies have reported that microbial-derived amyloids decrease the clearance of Aβ through promoting pyrin domain-containing protein 3 (NLRP3) inflammasome activation followed by microglial expansion and IL-1β/IL-18 secretion [55, 56]. Interestingly, extracellular vesicles (EVs) derived from gut bacteria are also related to the development of AD. For example, *Helicobacter pylori*-derived EVs can enter the brain and induce glial cell activation and neuronal impairment via complement component 3 (C3)-C3a receptor (C3aR) signaling, potentially accelerating AD development [57]. Furthermore, neurotoxic EVs derived from pathogens, such as *Paenalcaligenes hominis*, *Pseudomonas aeruginosa* and *Aggregatibacter actinomycetemcomitans*, embody components such as LPS that elicit a robust CNS immune response [58-60]. The reduction in the number of intestinal microfold cells induced by gut dysbiosis exacerbates AD through activating microglia, reducing the number of neurons, and increasing tau phosphorylation [61]. Gut dysbiosis can also directly influence synaptic and neuronal plasticity and modulate hippocampal learning processes [62]. In summary, gut dysbiosis directly or indirectly participates in AD development through multiple pathways, as shown in Figure 1.

## 2.2 Adipose tissue-brain crosstalk

### 2.2.1 Evidence for the involvement of adipose tissue disorders in AD development

Adipose tissue is extensively spread throughout the human body and is essential for regulating multiple physiological functions, such as energy balance, metabolism and immune responses, including white and brown adipose tissue [63]. Obesity is characterized by a body mass index of 30 kg/m^2^ or more and is often related to increased risk and adverse consequences for multiple disorders, including cardiovascular diseases, diabetes, and various cancer types [64]. Currently, accumulating evidence links obesity to the pathology and progression of AD [65, 66]. Obesity leads to adipose tissue remodeling, including adipose tissue expansion, secretory profile alteration, and immune and inflammatory landscape dysregulation [67]. Obesity can stimulate insulin resistance and diabetes, subsequently triggering cognitive impairment and the eventual development of AD [18].

Adipose tissue disorders caused by obesity participate in AD initiation and progression through complex crosstalk between adipose tissue and the brain. Clinical and animal evidence has revealed the relationship between obesity and AD. Extensive longitudinal studies and meta-analyses have confirmed that midlife obesity markedly increases dementia and AD risk [68-71]. In addition, a meta-analysis of observational studies revealed that middle-aged obesity was linked to a greater risk of AD [72]. Research has also revealed that AD prevalence increases exponentially when obesity is included in predictive models. Morys et al. performed an observational cohort study to investigate the role of obesity in cognitive dysfunction, and the results showed that obesity is associated with poor cognition [73]. In addition, obese individuals present decreased hippocampal and cortical volume, a faster rate of brain atrophy, and decreased cognition [74]. A series of animal experimental models have also shown that obesity promotes the occurrence and progression of AD. For instance, Ito et al. found that obese mouse-induced diabetic conditions exacerbated cognitive impairment in AD mice by inducing a specific tau phosphorylation pattern and increasing tau aggregation [75]. Mice fed a high-fat diet demonstrated weight gain related to decreased hippocampal neurogenesis, increased hippocampal Aβ production and deficits in cognitive tasks [76, 77].

### 2.2.2 The potential mechanisms of adipose tissue-brain crosstalk in the development of AD

The key mechanisms of adipose tissue disorders in AD etiology have recently drawn widespread focus, wherein the main alterations of obese adipose tissue include the occurrence of chronic inflammation, shifts in fatty acid release and the adipokine secretion profile, the dysregulation of immune cell infiltration, and adipose tissue-mediated metabolic disorders, which exert crucial roles in AD pathogenesis via regulating neuroinflammation, microglial activation and Aβ and tau metabolism [66].

Adipose tissue may influence AD pathogenesis and progression through several mechanisms. First, excessive exposure of immune cells in adipose tissue to free fatty acids mediates inflammatory cell activation and proinflammatory adipocytokine secretion [10]. Inflamed adipose tissue releases large amounts of signaling molecules and proinflammatory adipokines, such as IL-1β, IL-6, TNFα, cyclooxygenase 2 (COX2), inducible nitric oxide synthase (iNOS), NLRP3, insulin-like growth factor 1 (IGF-1), and chemerin [78-80]. These proinflammatory cytokines enter the circulation and cross the disrupted BBB into the brain to promote neuroinflammation by activating microglia and astrocytes and increasing the level of proinflammatory mediators in the brain, such as the hippocampus [81]. In addition, adipose tissue-resident macrophages favor proinflammatory M1 polarization, interacting with activated innate lymphoid cells (ILCs), neutrophils, T cells and dendritic cells to maintain a neuroinflammatory state through infiltration from the periphery into the CNS [82, 83]. The proportions of CD3+ T cells, CD8+ T cells, effector memory T cells, and CD69+ lymphocytes in the brains of obese AD mice are also greater than those in the brains of normal weight mice [84]. Central inflammation directly promotes Aβ deposition, disrupts existing neurons, reduces microglial phagocytosis of Aβ and prevents the proliferation and differentiation of new neurons, contributing to the development of AD [85].

**Figure 2.**
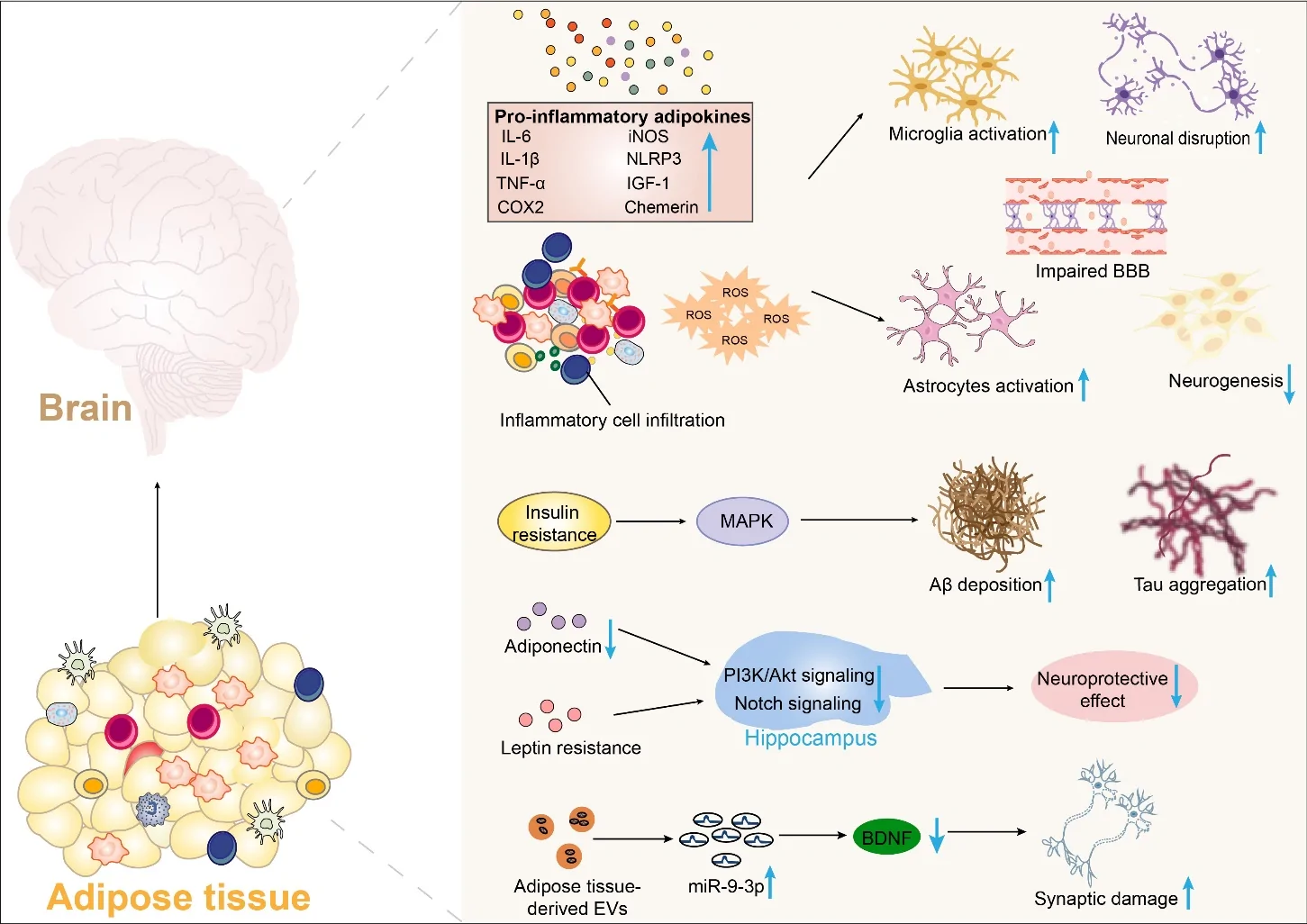
**Mechanisms of adipose tissue-brain crosstalk in the development of AD**. Adipose tissue disorders caused by obesity participate in AD initiation and progression through complex crosstalk between adipose tissue and the brain. For instance, obesity-associated adipose tissue dysfunction promotes inflammatory cell activation and the secretion of a variety of proinflammatory adipokines. These inflammatory cells and adipokines disrupt the BBB and enter the brain to promote microglia and astrocyte activation, disrupt neurons and impair neurogenesis. Obese adipose tissue promotes brain insulin resistance and induces Aβ deposition and tau aggregation via the MAPK signaling pathway. In addition, the neuroprotective effects of adiponectin and leptin are weakened due to obesity-induced reductions in adiponectin production and leptin resistance. In addition, obese adipose tissue-derived EVs and their cargo can migrate to the brain and target neurons, leading to synaptic damage.

Adipose tissue disorders in obesity promote brain insulin resistance and subsequently induce neurofibrillary tangle formation and Aβ aggregation through activating neuronal mitogen-activated protein kinase (MAPK) pathway [86]. Studies have shown that leptin and adiponectin can activate the PI3K/Akt pathway and Notch signaling in the hippocampus, thereby exerting neuroprotective effects [87, 88]. Adiponectin can also suppress microglial NLRP3 inflammasome activation, rescue the reduction in AMPK phosphorylation and prevent the nuclear translocation of NF-κB, subsequently inhibiting neuroinflammation [89, 90]. Therefore, obesity-induced leptin resistance and reduction in adiponectin production may promote AD progression. Wang et al. reported that EVs derived from the adipose tissue of obese mice induced synaptic damage, chronic neuroinflammation and cognitive impairment. Further mechanistic experiments revealed that EVs derived from adipose tissue and their cargo miR-9-3p can migrate to the hippocampus, leading to damage to synapses and cognitive decline through reducing brain-derived neurotrophic factor (BDNF) [91]. Increased adiposity increases reactive oxygen species (ROS) levels in the brains of mice, causing a decline in BDNF, a significant molecule that regulates neuronal plasticity and regeneration, and promoting a decline in cognitive function [92, 93]. These results indicate that adipose tissue disorders trigger a cascade of pathological events contributing to the pathogenesis of AD through mediating neuroinflammation, promoting brain insulin resistance, releasing EVs, and increasing ROS levels in the brain, as described in Figure 2.

## 2.3 Liver-brain crosstalk

### 2.3.1 Evidence for the involvement of liver dysfunction in AD development

A growing amount of evidence points to a connection between peripheral metabolic disorders and AD risk. Liver is a significant hub for multiple physiological processes and is mainly responsible for metabolic regulation and detoxification, immunomodulation, protein synthesis and lipid homeostasis [94]. Recently, the critical importance of liver-brain crosstalk in AD pathophysiology has received increasing attention. The liver is the major organ for clearing Aβ produced centrally and peripherally [95]. Aβ released from the brain binds to other molecules, such as high-density lipoprotein (HDL) particles, and is transported to the liver, where it is subsequently taken up by the liver through the low-density lipoprotein receptor-related protein-1 (LRP-1) expressed in hepatocytes [96, 97].

Recently, numerous studies have demonstrated the role of liver disorders in AD progression. Wang et al. reported that cirrhosis patients have higher plasma Aβ40 and Aβ42 levels than normal controls do. A previous study revealed that cirrhosis-associated hepatitis B virus (HBV) infection is also linked to AD development [98]. In a cohort study, researchers explored the associations between liver serum markers and AD. Increased aspartate aminotransferase (AST) to alanine aminotransferase (ALT) ratios and decreased ALT levels are strongly correlated with cognitive impairment [99]. Seo et al. found that nonalcoholic fatty liver disease (NAFLD) patients had an approximately four-fold greater risk of developing cognitive impairment [100]. Another population-based cross-sectional study reported that the multivariable adjusted odds ratio related to moderate-to-severe NAFLD was 1.88 [95% confidence interval (CI) = 1.01-3.50] for AD [101]. Emerging evidence has demonstrated that hepatitis virus infections may increase dementia risk [102]. According to a cohort study, the hazard ratio for dementia in patients with hepatitis C virus was 1.36 (95% CI = 1.27-1.42) [103]. In addition to human studies, animal research has investigated how liver dysfunction relates to AD. Kim et al. explored the effect of NAFLD on the pathology of AD by constructing a NAFLD mouse model. The results revealed that chronic NAFLD led to obvious pathological signs of AD in wild-type mice [19]. Zheng et al. reported that with the progression of AD in amyloid precursor protein/presenilin 1 (APP/PS1) mice, metabolic disorders, including those in energy, amino acid and nucleic acid metabolism, occur in the liver [104]. Collectively, these findings suggest that liver dysfunctions, such as cirrhosis, hepatitis virus infection, NAFLD, and liver enzyme changes, are closely associated with AD pathology.

### 2.3.2 The potential mechanisms of liver-brain crosstalk in the development of AD

**Figure 3.**
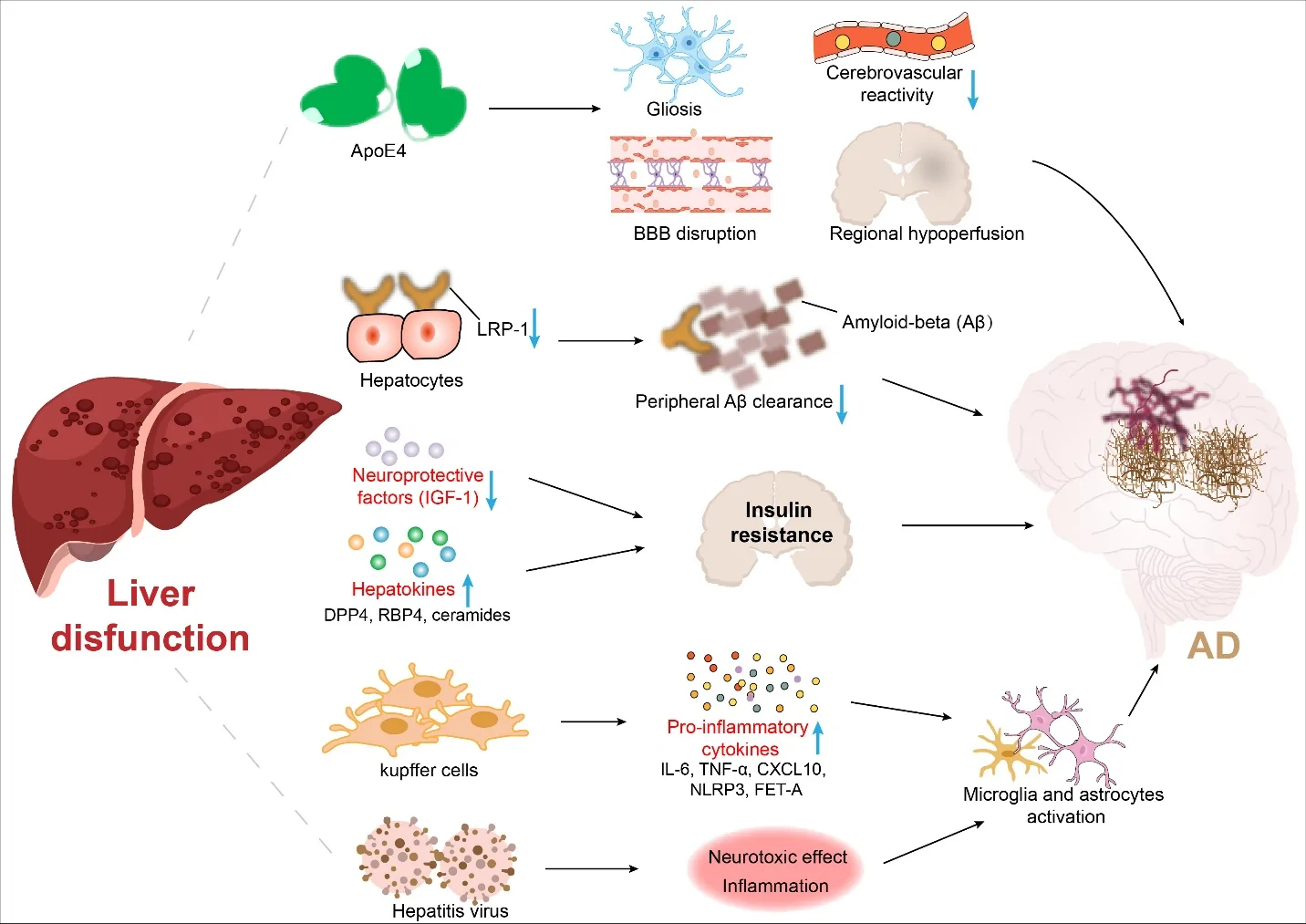
**Mechanisms of liver-brain crosstalk in the development of AD**. The underlying mechanisms of the crosstalk between the liver and brain involved in AD pathology are not fully understood, and imbalanced Aβ metabolism, impaired BBB integrity, neuroinflammation and toxin accumulation might play crucial roles. On the one hand, liver-expressed ApoE4 increases vessel-associated gliosis and impairs cerebrovascular functions, including inducing BBB disruption, reducing cerebrovascular reactivity and causing regional hypoperfusion, subsequently exacerbating brain amyloid pathology. On the other hand, diminished LRP-1 hinders hepatic Aβ clearance. Furthermore, increased secretion of hepatokines and decreased secretion of neuroprotective factors can promote brain insulin resistance, contributing to the progression of AD. Kupffer cells and hepatitis virus infections are also involved in AD neuropathology through promoting neuroinflammation.

The mechanisms of the liver-brain crosstalk involved in AD pathology remain largely unclear, and imbalanced Aβ metabolism, impaired BBB integrity, neuroinflammation and toxin accumulation might play crucial roles, as depicted in Figure 3. Apolipoprotein E (ApoE) mediates the elimination of soluble Aβ from the brain in an isoform-specific manner [105]. A large amount of evidence has confirmed the important role of ApoE in Aβ accumulation, efflux transport across the BBB, enzymatic degradation and as the carrier protein of plasma Aβ [106]. There are three major alleles of ApoE, namely, ApoE2, ApoE3, and ApoE4, of which ApoE4 is the primary risk factor for AD [107]. Over 75% of the body’s ApoE is generated in the liver, and the remainder is generated from the brain and macrophages [108]. Liu et al. explored the role of liver-derived ApoE in AD pathogenesis by constructing mouse models that specifically express ApoE in the liver. They reported that liver-expressed ApoE4 increased vessel-associated gliosis and impaired cerebrovascular functions, including inducing BBB disruption, reducing cerebrovascular reactivity and causing regional hypoperfusion, subsequently exacerbating brain amyloid pathology [109]. In the liver, Aβ is absorbed and eliminated by hepatocytes through binding to LRP-1 on the surface of hepatocytes [96]. Recently, Chandrashekar et al. reported that LRP-1 expression was reduced in mice with liver dysfunctions, such as alcohol-mediated liver injury and NAFLD, leading to reduced hepatic clearance of Aβ and increased Aβ levels in the brain [19]. NAFLD has also been reported to lead to a decrease in the secretion of neuroprotective factors such as IGF-1 and an increase in the release of hepatokines such as dipeptidyl peptidase 4 (DPP4), retinol binding protein 4 (RBP4) and ceramides, which causes insulin resistance in the CNS and promotes the progression of AD [110, 111].

During the development of hepatic disorders, resident Kupffer cells in the liver transform into the M1 proinflammatory phenotype and secrete multiple proinflammatory cytokines, like IL-6, TNF-α, C-X-C motif chemokine ligand 10 (CXCL10), NLRP3, lipocalin 2 and fetuin-A (FET-A), which can enter the brain and cause neuroinflammation [112]. Emerging evidence suggests that hepatitis virus infections might be involved in AD neuropathology via mediating direct neurotoxic effects, promoting systemic and/or cerebral inflammation and activating microglia and so on [113]. Epoxyeicosatrienoic acids (EETs) are key substances involved in maintaining homeostasis and are regulated mainly by soluble epoxide hydrolase (sEH) [114]. 14,15-EET can quickly pass through the BBB and regulate brain Aβ metabolism. Notably, the activity of liver sEH increases during aging, which induces a decrease in the level of 14,15-EET in plasma and subsequently contributes to Aβ deposition in the brain [11]. Another hypothesis for the relationship between liver dysfunction and AD arises from reduced albumin synthesis. Liver dysfunction, such as cirrhosis, triggers a notable reduction in the synthesis of albumin, the main carrier protein of Aβ in the circulation, thereby promoting an increase in plasma Aβ levels [98]. Nevertheless, the underlying mechanisms of liver dysfunction in the development of AD remain incompletely clear, and additional research is necessary to elucidate the specific molecular mechanisms involved.

## 2.4 Bone-brain crosstalk

### 2.4.1 Evidence for the involvement of bone disorders in AD development

In general, the skeleton is a dynamic and complex system that is constantly renewing and remodeling in response to an ever-changing environment. Bone homeostasis is orchestrated mainly by osteoblasts, osteocytes and osteoclasts [115]. In addition, bone marrow mesenchymal stem cells and the bone microenvironment, which includes inflammatory, endothelial, and Schwann cells, might help maintain bone homeostasis [116]. Recently, increasing evidence has shown that AD and orthopedic conditions, like osteoarthritis and osteoporosis, frequently coexist and are closely linked in pathogenesis [117]. Bone tissue and the brain can regulate each other in a variety of ways through the bone-brain axis, thus influencing AD development [118]. An association between bone tissue and the brain has been reported in a series of clinical and animal studies.

In clinical studies, increasing evidence has shown that bone disorders like osteoporosis, osteoarthritis and rheumatoid arthritis are linked to AD [119]. A longitudinal follow-up study indicated that osteoporosis increased AD risk in adults aged ≥40 years [120]. Amouzougan et al. performed a cross-sectional observational study to explore the influence of postmenopausal osteoporosis on AD or other dementias. The analysis revealed that AD prevalence was greater in postmenopausal females suffering from severe osteoporosis [121]. Further research has uncovered that osteoporosis is correlated with dementia in both sexes [122]. Osteoarthritis has also been reported to be strongly linked to AD risk. For example, a cross-sectional study of 21,982 adults showed a notably increased AD risk in people with osteoarthritis, especially those accompanied by pain [123]. With the intensification of chronic pain linked to osteoarthritis, there is a corresponding increase in the risk of AD [124]. Du et al. conducted a follow-up study in senior populations and reported that osteoarthritis was significantly related to accelerated Aβ accumulation and greater deposition of tau in the brain [118]. Furthermore, rheumatoid arthritis is also linked to AD. In clinical studies, rheumatoid arthritis patients have obviously decreased cognitive function and an increased incidence of dementia due to AD compared with healthy controls [125, 126]. An expanding body of basic evidence has revealed that bone disorders are tightly connected to the onset of AD. For example, compared with healthy control mice, knee osteoarthritis mice exhibit more significant pathological changes associated with AD [127].

### 2.4.2 The potential mechanisms of bone-brain crosstalk in the development of AD

**Figure 4.**
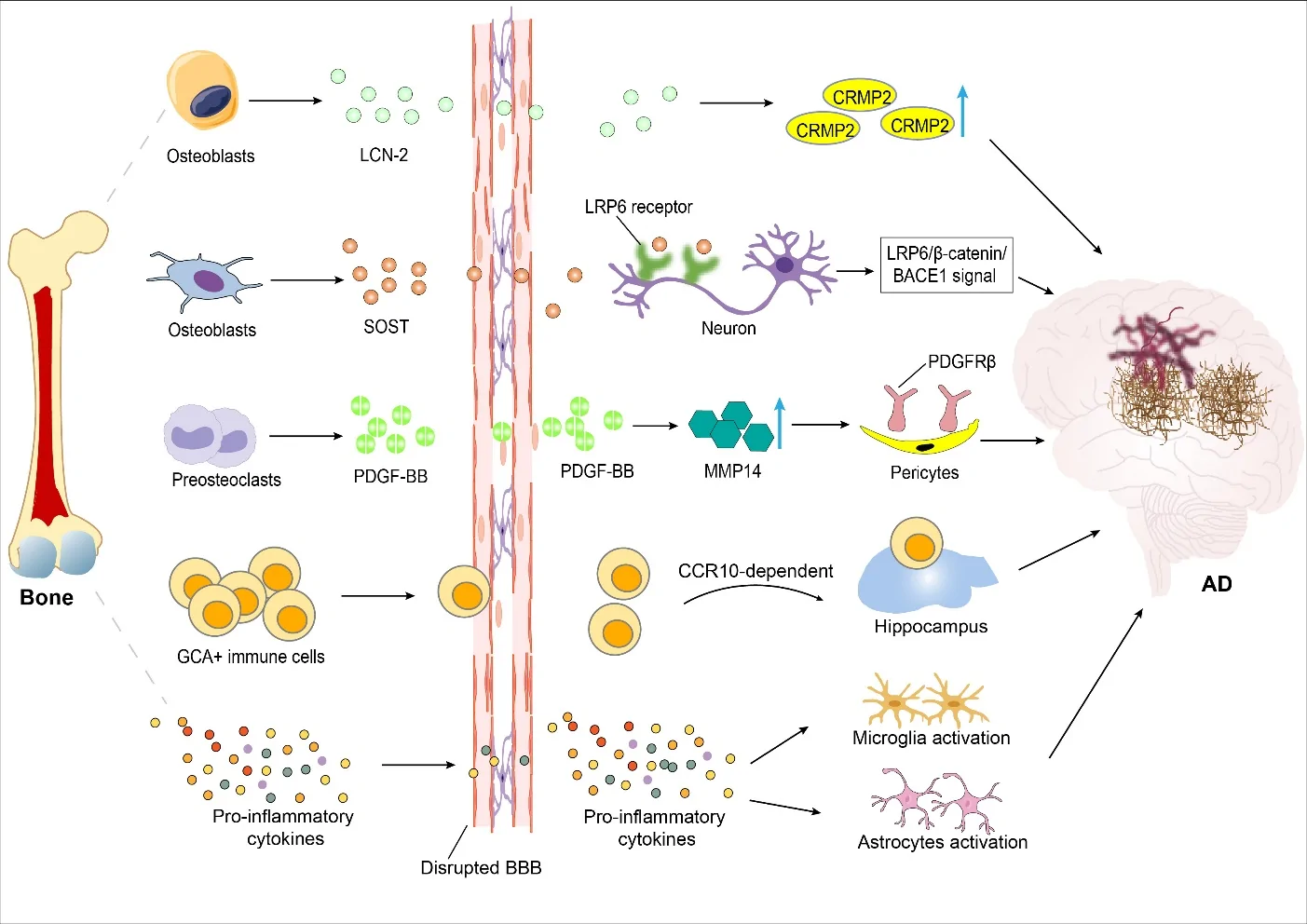
**Mechanisms of bone-brain crosstalk in the development of AD**. Bone-derived proteins, such as LCN-2, SOST and PDGF-BB are involved in AD development through different pathways. osteoblast-derived LCN-2 promoted AD development through increasing the level of hippocampal CRMP2. SOST can cross the BBB and bind to the low-density- LRP6 of neurons, leading to dysfunction of the Wnt/β-catenin pathway, which further increases Aβ production via LRP6/β-catenin/BACE1 signaling. PDGF-BB secreted by bone can promote the shedding of PDGFRβ from the surface of pericytes through the upregulation of MMP14, thereby inducing hippocampal BBB impairment. Moreover, bone marrow-derived circulating GCA+ immune cells can invade the cortex and hippocampus and promote the development of AD. Peripheral proinflammatory factors produced by bone cross the damaged BBB into the brain and promote the activation of microglia and astrocytes, contributing to AD pathology.

The underlying mechanisms of bone disorders in the pathogenesis of AD involve multiple signaling pathways, which include mainly bone-derived proteins, bone-derived immune cells, inflammation and calcium imbalance. Lipocalin-2 (LCN-2), which is secreted by osteoblasts, participates in multiple pathophysiological processes, including inflammation, innate immunity, apoptosis and metabolism [128]. Wu et al. reported that LCN-2 expression in osteoblasts was signiﬁcantly increased in an AD mouse model. Further experiments revealed that osteoblast-derived LCN-2 promoted AD development through increasing the level of hippocampus collapsin response mediator protein 2 (CRMP2), a mediator that affects synaptic plasticity and promotes abnormal phosphorylation of the tau protein [129]. Sclerostin (SOST), another protein secreted by mature bone cells, can inhibit Wnt-β-catenin signaling through binding with low-density-lipoprotein receptor 5/6 (LRP5/6) [130, 131]. Clinically high blood SOST levels are correlated with cognitive impairment and brain Aβ burden in aged people and AD sufferers [132]. Shi et al. demonstrated that SOST secreted by osteocytes can traverse the BBB of mice and bind with the LRP6 receptor of neurons, leading to impairment of Wnt/β-catenin signaling, which further increases Aβ production through LRP6/β-catenin/BACE1 signaling [12]. Platelet-derived growth factor-BB (PDGF-BB) is an important regulator of angiogenesis that promotes the formation and stability of blood vessels by acting on endothelial cells and surrounding smooth muscle cells [133]. Research has shown that PDGF-BB secreted by bone can promote the detachment of PDGF receptor *β* (PDGFR*β*) from pericytes through the upregulation of matrix metalloproteinase 14 (MMP14), thereby inducing hippocampal BBB impairment and memory deficits [134]. Hippocampal BBB impairment and cognitive dysfunction mediated by bone-derived PDGF-BB might promote AD development. Zhou et al. reported that bone marrow-derived circulating grancalcin (GCA^+^) immune cells are related to AD and can invade the cortex and hippocampus through CCR10 signaling. Subsequently, GCA in the brain further competitively binds to the microglial LRP1 receptor, consequently hindering the phagocytosis and removal of Aβ while exacerbating neuropathological alterations [135]. Peripheral proinflammatory factors produced by orthopedic diseases cross the damaged BBB into the brain and promote the transformation of microglia and astrocytes into proinflammatory phenotypes, thus producing many neuroinflammatory factors in the brain [136]. The detailed mechanisms by which bone disorders promote AD progression are shown in Figure 4.

### 2.5 Other types of inter-organ crosstalk

In addition to the organ disorders discussed above, other organ disorders, such as pulmonary diseases, cardiovascular diseases, kidney dysfunction, and periodontal disease, play important roles in AD onset [137-140]. A large prospective community-based study found that impaired lung function and lung disease increased susceptibility to dementia and mild cognitive impairment [137]. Baranova et al. conducted a Mendelian randomization study to explore the causal relationship linking COVID-19 to AD. The results indicated that severe COVID-19 increased AD risk [141]. Changes in the respiratory microbiome caused by lung disease, through the release of LPS or outer membrane vesicles carrying bacterial toxins, lead to BBB damage, activation of glial cells, and infiltration of proinflammatory factors, thus contributing to AD [142]. The brain and heart also influence each other through a still poorly understood brain-heart axis. Accumulating evidence suggests that cardiovascular diseases like heart failure and coronary heart disease are linked to an increased risk for AD [143]. Cardiac output reduction, inflammation, neurohormonal activation and microvascular dysfunction induce cerebral blood flow reduction and hypoxia, subsequently promoting the development of AD [144]. Emerging experimental and clinical studies have also shown that kidney dysfunction contributes to dementia and AD onset [139, 145]. Multiple clinical studies have shown that individuals with chronic kidney disease (CKD) are more susceptible to cognitive decline and AD [146]. Mechanistic studies have revealed that CKD-related increases in renin-angiotensin system activation, uremic toxin accumulation, and endothelin secretion can induce BBB damage, glial cell activation, oxidative stress, and neurotoxicity [147]. Periodontal disease is a chronic inflammatory disorder mediated by microbial dysbiosis that impacts AD via the gut-brain axis [148]. The pathogenesis of AD is complex and involves multiple types of inter-organ crosstalk, and further exploration of the contributions of various organ disorders to AD onset is needed.

## 3. Prospects of inter-organ crosstalk in AD therapy

Given the tight connection between extracerebral organ disorders and AD, treatments targeting brain-centered inter-organ crosstalk are emerging. Gut dysbiosis induced by intestinal disorders is involved in AD pathogenesis via gut-brain crosstalk, suggesting that manipulating the gut microbiota with antibiotics or probiotics may be effective in treating AD [149]. Some antibiotics may have beneficial effects on AD through attenuating neuroinflammation caused by gut dysbiosis. For example, rifampicin administration reduces the levels of Aβ, as well as oxidative and inflammation-related cytokines in the brains of AD mice [150]. In AD mice, the mTOR inhibitor rapamycin has been shown to enhance cognition, lower Aβ levels, reduce tau protein phosphorylation, and diminish microglial activation [151, 152]. Similarly, minocycline has similar effects in an AD mouse model, including reducing microglial activation, inflammation, and Aβ levels and improving cognition [153]. How probiotic supplementation affects cognitive function has been explored via clinical trials in both elderly individuals and patients with AD. The findings indicate that probiotic supplementation might improve cognitive performance [154, 155]. Accumulating evidence has also shown an association between adipose tissue disorders such as obesity and AD. Obesity treatment or prevention may be a safe and efficacious approach for treating AD. Adiponectin is a benign adipokine, and its secretion is reduced in obese individuals [156]. Clinical and animal studies have shown that the administration of adiponectin or adiponectin receptor homologs can alleviate inflammation, reduce Aβ and tau protein aggregation, stimulate neurogenesis and improve cognition [157]. Interventions for obesity, such as exercise training and caloric restriction, may also have the potential for preventing and treating AD. A series of studies reported that physical exercise was related to reduced Aβ deposition, decreased inflammation and microglial activation, increased brain-derived neurotrophic factor and cognitive function [158-160]. Glucagon-like peptide-1 (GLP-1), a secreted hormone in the gut, has attracted increasing interest as a potential factor linking metabolic disorders to brain dysfunction [161]. GLP-1 receptor agonists such as semaglutide and liraglutide are widely utilized for managing diabetes and obesity [162]. Several studies have reported that the capacity of liraglutide to alleviate AD-associated pathological alterations, including Aβ deposition, tau hyperphosphorylation, and loss of neurons and synapses, is accompanied by cognitive improvement [163]. Wang et al. found that semaglutide decreased Aβ plaque and neurofibrillary tangle accumulation, promoted glucose metabolism in the brain and improved cognition in AD mice [164]. A few clinical trials have investigated the influence of dietary interventions, physical exercise, or GLP-1 receptor agonists on cognition in aging populations or elderly individuals with mild cognitive impairment, and the results revealed that these interventions improved cognition [165-167].

**Table 2:** Potential therapeutic approaches targeting brain-centered inter-organ crosstalk.

Therapeutic interventions	Targeted axis	Effects
**Rifampicin**	Gut-brain	Reduced Aβ Anti-oxidative Anti-inflammatory
**Rapamycin**	Gut-brain	Reduced Aβ Reduced tau phosphorylation Reduced microglia activation Improved cognition
**Minocycline**	Gut-brain	Reduced Aβ Reduced microglia activation Anti-inflammatory Improved cognition
**Adiponectin** **Adiponectin receptor homologs**	Adipose tissue-brain	Reduced Aβ Reduced tau aggregation Increased neurogenesis Improved cognition
**Physical exercise**	Adipose tissue-brain	Reduced Aβ Reduced microglia activation Anti-inflammatory Increased brain-derived neurotrophic factor Improved cognition
**Liraglutide**	Adipose tissue-brain	Reduced Aβ Reduced tau phosphorylation Decreased neuronal loss Decreased synaptic loss Improved cognition
**Semaglutide**	Adipose tissue-brain	Reduced Aβ Improved glucose metabolism Improved cognition
**Fluvastatin**	Liver-brain	Reduced Aβ Increased Aβ clearance
**Osteocalcin**	Bone-brain	Decreased neuronal apoptosis

Abbreviations: AD: Alzheimer's disease; Aβ: amyloid beta.

Current studies have provided an increasingly deep understanding of the contribution of liver dysfunction to AD pathogenesis, and there is a growing trend in research to explore liver-targeted therapies to prevent or treat AD. Liver-expressed LRP-1, a major mediator involved in the clearance of toxic Aβ in the brain and body, is considered a prospective target for treating AD [97]. For instance, research showed that fluvastatin enhances Aβ clearance and reduces its accumulation in an AD transgenic mouse model by increasing the expression level of LRP-1 in the BBB [168]. Potential treatments for AD targeting bone-brain axis are also emerging. Vitamin D supplementation can reduce Aβ deposition and oxidative stress and enhance cognition in AD patients and mouse models [169]. In addition, bone-secreted proteins such as osteocalcin delay cognitive decline through preventing neuronal apoptosis in the hippocampus [170]. Thus, therapies that target the crosstalk between peripheral organs such as the intestine, adipose tissue, liver, bones, and brain may be an emerging and effective treatment for AD.

Overall, strategies targeting brain-centered inter-organ crosstalk to treat AD have been widely explored in preclinical and clinical studies and are summarized in Table 2.

## 4. Conclusions

In recent decades, organ-organ crosstalk has allowed the exchange of messages and information, which is essential for maintaining homeostasis and coordinating organ functions. Increasing evidence indicates that AD is a multi-organ disease and that various peripheral organ dysfunctions can affect the occurrence and development of AD through inter-organ crosstalk, such as gut-brain crosstalk, adipose tissue-brain crosstalk, liver-brain crosstalk and bone-brain crosstalk. Despite decades of in-depth research, current treatments for AD are limited. Hence, exploring the mechanisms of peripheral organ disorders in the pathogenesis of AD from a multi-organ perspective will help develop more novel and effective therapies for AD. As highlighted in this review, we emphasize the current mechanisms of the inter-organ crosstalk between the brain and peripheral organs involved in the pathogenesis of AD, such as gut dysbiosis induced by intestinal disorders, which promotes AD development via the secretion of a variety of metabolites, functional byproducts and toxins; dysfunction of adipose tissue in obesity is involved in AD pathogenesis via changes in fatty acid and adipokine secretion profiles and metabolism; liver dysfunction mediates Aβ clearance reduction and toxin accumulation in the brain; and dysregulated bone tissue-derived proteins and immune cells, as well as calcium imbalance, accelerate AD development. However, the potential mechanisms involved in various peripheral organ disorders in AD pathogenesis have still not been fully clarified, and additional foundational studies are needed to explore the mechanisms involved. Furthermore, developing more promising inter-organ crosstalk-based therapeutic interventions for AD will provide more personalized and effective therapeutic options.

In conclusion, the crosstalk between the brain and peripheral organs may play an indelible role in AD development. A better understanding of the pathogenic “crosstalk” between the brain and extracerebral organs will help identify risk factors related to AD onset and progression, as well as explore novel therapeutic targets. In addition, further studies are needed to elucidate the role of interactive networks in the pathogenesis of AD, as each extracerebral organ not only affects brain function through the extracerebral organ-brain axis but also may act through cross-pathways. For instance, gut dysbiosis can disrupt the normal function of the bone and liver, thereby forming a complex, multi-factorial system that synergistically contributes to the onset and progression of AD. It is essential to explore comprehensive, dynamic, and personalized approaches for the treatment of AD tailored to the distinct inter-organ crosstalk profiles of individual patients.
